# Serum Pyridoxal 5′-Phosphate and Pyridoxic Acid Ratio Index with Prognosis of Colorectal Cancer: A Prospective Cohort Study

**DOI:** 10.3390/nu16213685

**Published:** 2024-10-29

**Authors:** Xue Li, Lei Xu, Qing-Jian Ou, Huan Xu, Yuan-Yuan Chen, Yu-Jing Fang, Cai-Xia Zhang

**Affiliations:** 1 Department of Epidemiology, School of Public Health, Sun Yat-sen University, Guangzhou 510080, China; 2 State Key Laboratory of Oncology in South China, Guangdong Provincial Clinical Research Center for Cancer, Sun Yat-sen University Cancer Center, Guangzhou 510060, China; 3 Chronic Noncommunicable Disease Prevention and Control Department, Guangzhou Center for Disease Control and Prevention, Guangzhou 510440, China

**Keywords:** pyridoxal 5′-phosphate, PAr, colorectal cancer, survival, cohort study

## Abstract

Background: Studies on the association between serum vitamin B_6_ status and colorectal cancer prognosis are limited and have yielded inconsistent results. This study investigated the association of pyridoxal 5′-phosphate (PLP) and pyridoxic acid ratio (PAr) index with colorectal cancer survival. Methods: A total of 1286 colorectal cancer patients diagnosed since 2010 were selected from the Guangdong Colorectal Cancer Cohort study. Serum levels of PLP, pyridoxal, and 4-pyridoxic acid were measured using ultra-high-performance liquid chromatography–tandem mass spectrometry. The study followed overall mortality and colorectal cancer-specific mortality until December 2023. Multivariable Cox proportional hazards regression models were applied to calculate hazard ratios (HRs) and 95% confidence intervals (95% CIs). Restricted cubic spline and stratified analysis were performed. Results: During a median follow-up of 77.36 months, 331 deaths were recorded, with 293 specifically attributed to colorectal cancer. Higher PLP levels were associated with a longer overall survival (HR_Q4 vs. Q1_, 0.63; 95% CI: 0.46, 0.87; *p* for trend = 0.008) and colorectal cancer-specific survival (HR_Q4 vs. Q1_, 0.62; 95% CI: 0.44, 0.87; *p* for trend = 0.006). Non-linear associations were observed between serum PLP and overall and colorectal cancer-specific survival (*p* for non-linear < 0.05). However, PAr was not significantly associated with either overall survival (HR_Q4 vs. Q1_, 1.03; 95% CI: 0.75, 1.41) or colorectal cancer-specific survival (HR_Q4 vs. Q1_, 1.01; 95% CI: 0.72, 1.42). The association between serum PLP and both overall survival and colorectal cancer-specific survival (*p* for interaction < 0.05) varied by alcohol drinking status. Conclusions: Higher serum PLP levels, but not PAr, may be associated with improved overall and colorectal cancer-specific survival.

## 1. Introduction

Colorectal cancer is a primary cause of cancer-related morbidity and mortality worldwide [1,2]. The mortality rates for colorectal cancer are on the rise globally [3,4]. From 1990 to 2019, the number of deaths from colorectal cancer increased from 518,126 to 1.09 million [2]. The 5-year survival rate for metastatic colorectal cancer is 14% [5]. Improving long-term outcomes for colorectal cancer patients remains a significant challenge. As cancer cells rely on metabolites from the patient’s diet [6], identifying modifiable factors, such as diet, is essential to prevent mortality in individuals with colorectal cancer [7,8,9,10].

Vitamin B_6_, mainly obtained through the diet, plays a crucial role in regulating and participating in significant processes such as carbohydrate metabolism, DNA synthesis, and one-carbon cycling, acting as a cofactor and essential nutrient [6,11,12,13,14,15,16]. Blood measurements of B vitamins can overcome the limitation of relying on self-reported dietary intake and may provide a more accurate assessment of exposure, as the bioavailability of serum B vitamins may be compromised [17]. Pyridoxal 5′-phosphate (PLP), the biologically active form of vitamin B_6_, is commonly utilized as the primary indicator of overall vitamin B_6_ status in the body [18]. The pyridoxic acid ratio (PAr) index, defined as the ratio of 4-pyridoxic acid (PA) over the sum of PLP and pyridoxal (PL), serves as a marker of increased vitamin B_6_ catabolism during inflammation and related cellular immune activation [19]. The PAr is significantly associated with inflammatory markers such as C-reactive protein (CRP), an acute phase marker, and kynurenine/tryptophan ratio (KYN/TRP), which is linked to cellular immunity [20]. Inflammation-related processes may lead to an imbalance in the plasma concentrations of B_6_ vitamers, causing an increase in PA relative to PL + PLP and thus resulting in an elevated PAr [21]. Albumin, the primary carrier of PLP, is often found at reduced levels during inflammation, while increased activity of membrane-bound phosphatases, such as alkaline phosphatase, facilitates the uptake of PLP into tissue. Moreover, oxidative stress can upregulate enzymes involved in aldehyde metabolism, such as aldehyde oxidase (AOX) and aldehyde dehydrogenases, which convert PL to PA. Progressive kidney damage due to chronic inflammation may also increase plasma PA levels in comparison to PLP and PL [21]. Additionally, the relationship between the acute inflammatory response—evidenced by markers like CRP—and the redistribution of PLP from plasma to other tissues suggests a connection between elevated PAr and various inflammatory processes [22]. Therefore, PAr serves as a marker for increased vitamin B_6_ catabolism during inflammation and related cellular immune activation.

Previous studies have specifically focused on PLP and PAr in relation to colorectal cancer risk [23,24,25,26] and, to a lesser extent, survival [27,28,29,30,31]. The findings regarding the association between PLP levels and colorectal cancer survival have been inconsistent [28,30,31]. One study indicated that elevated plasma PLP levels were associated with a better prognosis for colorectal cancer patients, while two other studies found no significant association. To date, only one study has investigated the relationship between PAr and colorectal cancer survival, reporting a positive association [30]. Therefore, the epidemiological evidence on the protective role of serum PLP against colorectal cancer is inconclusive, and research on PAr and colorectal cancer survival is limited to a single study conducted on European populations.

Studies examining the relationship between circulating vitamin B_6_ concentrations and colorectal cancer prognosis are limited in China. This study aims to prospectively investigate the association of serum PLP and PAr with survival in Chinese colorectal cancer patients. The hypothesis was that higher serum PLP levels and lower PAr are associated with improved colorectal cancer survival.

## 2. Materials and Methods

### 2.1. Study Population

**Figure 1:**
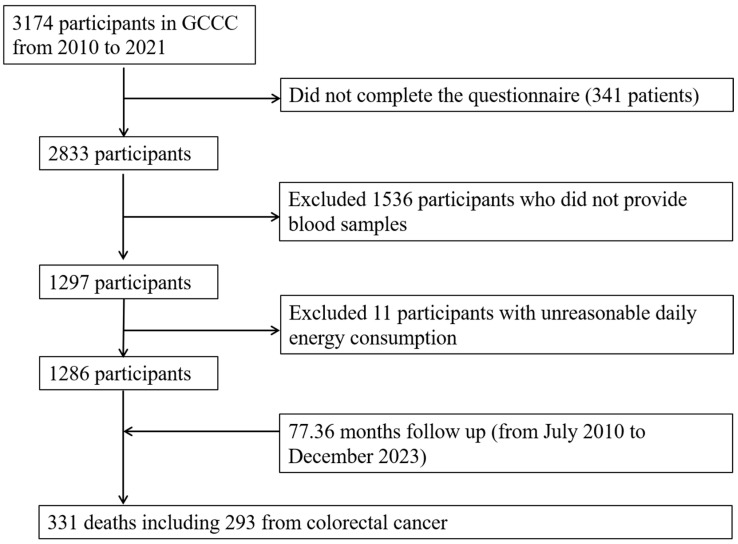
Flow chart of study participants. Abbreviations: GCCC, Guangdong Colorectal Cancer Cohort study.

This study utilized data from the Guangdong Colorectal Cancer Cohort (GCCC), an ongoing prospective cohort study aimed at evaluating genetic and environmental factors influencing the survival of individuals diagnosed with colorectal cancer. The cohort was established in July 2010 at the Sun Yat-sen University Cancer Center in Guangzhou, China [32,33]. Potential colorectal cancer patients were screened for eligibility, and subsequent enrollment in the cohort was conducted according to the following inclusion criteria: aged 30–75 years, with a histological diagnosis of primary colorectal cancer (International Classification of Diseases, version 10 [ICD10/C18-C20]) within 3 months of diagnosis, and either being native to Guangdong or having maintained residence in Guangdong for a minimum of 5 years. Patients were not eligible to participate if they had a diagnosis of another type of cancer or if they were unable to communicate or understand Mandarin or Cantonese effectively. A total of 3174 cases were identified for the period between July 2010 and May 2021. Of the aforementioned cases, 2833 were eligible and thus underwent an interview, resulting in a response rate of 89.26%. A total of 1297 blood samples were available and detected from among eligible cases (Figure 1). After excluding 11 cases with unreasonable daily energy consumption information, the final analysis encompassed 1286 cases.

### 2.2. Blood Collection and Biochemical Measurements

On the second day of their hospitalization, each participant provided a fasting venous blood sample of approximately 5 mL. All participants had not received any treatment or surgical operation before the blood collection. Upon arrival at the laboratory, the blood samples were subjected to centrifugation at 3000 rpm for a period of 10 min at a temperature of 4 °C. Subsequently, an aliquot of each serum sample was transferred into eight 200 μL tubes and immediately frozen at −80 °C until analysis was initiated.

Serum vitamin B_6_ species, comprising PLP, PL, and PA, were measured using the ultra-high-performance liquid chromatography–tandem mass spectrometry (UHPLC-MS/MS) method, as exemplified in previous studies [34,35]. The detection process was performed with an Agilent 1290 UHPLC system in conjunction with an Agilent 6495 triple quadrupole instrument (Agilent, Santa Clara, CA, USA). The precise methodology employed has been previously described in detail [36,37]. The recovery rates of serum vitamin B_6_ ranged between 85% and 118%, with mean inter-batch coefficients of variation at 11.4% for PLP, 8.1% for PL, and 10.1% for PA, respectively.

### 2.3. PAr Definition

The calculation of PAr involves the division of PA by the sum of the PLP and PL. The procedures for quantifying PA, PL, and PLP concentrations are delineated in the preceding section.

### 2.4. Outcome Ascertainment and Follow-Up

In this study, two primary outcomes were evaluated: overall survival and colorectal cancer-specific survival. Overall survival considered death from any cause, while colorectal cancer-specific survival focused on deaths due to colorectal cancer. Overall survival was defined as the time elapsed between the pathological diagnosis of colorectal cancer and the occurrence of any cause of death. Colorectal cancer-specific survival was defined as the time elapsed between the pathological diagnosis of deaths specifically attributed to colorectal cancer. Participants who did not experience an event of interest within the study period were considered to have been censored on the date of the final follow-up evaluation. Follow-up commenced at the time of colorectal cancer diagnosis and continued until a cancer-related event occurred or the last outcome ascertainment on 1 December 2023. The data pertaining to mortality among participants was gathered on a regular basis through a multifaceted approach, encompassing the reporting system overseen by the Guangzhou Center for Disease Control and Prevention, medical records, and active follow-up through telephone interviews with patients or their next-of-kin. Physicians identified colorectal cancer-specific causes of death based on ICD-10 codes.

### 2.5. Covariate Ascertainment

At baseline, patient information on demographics, lifestyle factors, dietary habits, and clinical data was obtained through in-person interviews or extracted from medical records [38]. Demographic data included sex, age, marital status, and average monthly income. Lifestyle habits covered smoking status, alcohol consumption, and physical activity.

A validated food frequency questionnaire (FFQ) was employed to ascertain dietary information [38]. Trained interviewers posed inquiries to the participants in a personal face-to-face interview. They requested that the participants estimate the frequency (never, per year, per month, per week, per day) and amount of each food item consumed. The average daily nutrient intake was calculated by multiplying the frequency and portion size of each food item by its nutrient content based on the China Food Composition Table 2002 [39]. Participants were provided with photographs of standard food portions to aid in estimating their intake. The residual method was employed for the purpose of adjusting the dietary intake of nutrients in consideration of energy intake (in kcal/d) [40]. Clinical factors, such as age at diagnosis, cancer stage (I, II, III, and IV), tumor site (colon, rectum), degree of differentiation (well-differentiated, moderately differentiated, poorly differentiated), surgical operation (yes or no), and administration of radiotherapy or chemotherapy (yes or no), were extracted from hospital medical records.

A smoker was defined as someone who had smoked at least one cigarette per day for more than 6 months consecutively or cumulatively in their lifetime. The patient’s smoking status was classified as either “never-smoking” or “ever-smoking” based on the criteria that they had smoked continuously or cumulatively for a period of more than six months. In this categorization, ever-smoking included both current and former smoking. Regular drinking was defined as consuming alcohol at least once per week during the past year. Physical activity levels were assessed based on self-reported occupational, household, and leisure-time activities. The mean metabolic equivalent task (MET)-hours value for each activity was obtained by averaging all comparable activities in the Compendium of Physical Activities [41,42]. The MET-h/week for each subject was calculated according to the following formula: number of days/week × hours per day × MET for a specific activity. Body mass index (BMI) was calculated using the following formula: weight (kg)/height squared (m^2^).

### 2.6. Statistical Analysis

In cases where data exhibited abnormal detection patterns, such as values exceeding the upper limit of the quantitative standard curve, the median value was substituted. Baseline characteristics of the study population were presented according to sex-specific quartiles of serum PLP. Continuous variables were expressed as mean (standard deviation) or median (*P*_25_, *P*_75_), while categorical variables were presented as frequencies (percentages). Differences between groups were assessed using one-way ANOVA or Kruskal-Wallis tests for continuous variables and chi-square tests for categorical variables.

Cox proportional hazards regression models were fitted to evaluate hazard ratios (HRs) and 95% confidence intervals (CIs) to examine the association of serum PLP and PAr with survival outcomes. No violations of the proportional hazard assumption were detected. In the multivariable analyses, adjustments were made for potential confounding factors assessed at study recruitment. Model 1 adjusted for age (continuous), sex (male, female), BMI (continuous), smoking (ever or never), drinking (regular or never), MET (continuous), total energy intake (continuous), and protein intake (continuous). Model 2 included additional adjustments for differentiation (well-differentiated, moderately differentiated, poorly differentiated), surgery (yes or no), radiotherapy or chemotherapy (yes or no), and history of cancer in first-degree relatives (yes or no). A linear trend was tested using the median value for each categorical variable (Q1-Q4) as a continuous variable in Cox proportional hazards modes. Restricted cubic spline (RCS) analysis with 4 knots was used to evaluate dose–response relationships and potential non-linear associations between serum vitamin B_6_ and survival outcomes. The likelihood ratio test was employed to examine non-linearity.

Stratified analysis was performed by sex, age, BMI, smoking status, cancer stage, and tumor site. Additionally, given the potential influence of alcohol on vitamin B_6_ absorption and one-carbon metabolism [43], stratified analyses were also conducted according to alcohol drinking status. Interaction tests were performed by comparing multivariate models with and without interaction terms using cross-product terms. The *p*-value for interaction was derived from the log-likelihood statistic. To examine the robustness of the result, two sensitivity analyses were performed: (1) additional adjustment for dietary vitamin B_6_ intake based on model 2; (2) additional adjustment for the timing of blood-sample collection based on model 2.

All statistical analyses were performed using R software, version 4.4.0 (R Project for Statistical Computing). *p*-values < 0.05 were considered statistically significant based on two-sided tests.

## 3. Results

### 3.1. Characteristics of Participants in GCCC Study

The median (*P*_25_, *P*_75_) serum PLP concentration and PAr for these 1286 colorectal cancer patients was 6.03 (3.21–11.10) nmol/L and 0.69 (0.49–1.00), respectively. A median follow-up period of 77.36 months yielded a total of 331 deaths among the 1286 colorectal cancer patients, with 293 of these deaths attributed specifically to colorectal cancer. Among the patients who died, 218 (30.24% out of 721) were male and 113 (20.00% out of 565) were female. The median (*P*_25_, *P*_75_) age at enrollment was 58.53 (49.98–65.03) years, and 721 (56.07%) of the patients were men. Table 1 presents the baseline characteristics of the 1286 colorectal cancer patients, categorized by quartile of serum PLP. A statistically significant difference was observed in drinking status among patients with varying serum PLP levels (*p* = 0.017). There was also a significant difference in cancer stage based on serum PLP levels (*p* < 0.001). Additionally, significant differences were noted in other serum vitamin levels across varying serum PLP quartiles (all *p* < 0.05). Significant differences were observed in total energy (kcal/day) and protein (g/day) intake across the various groups (both *p* < 0.05), while significant differences were not noted in dietary B vitamin intake levels across varying serum PLP quartiles (all *p* > 0.05).

### 3.2. Association of Serum PLP and PAr with Survival

The concentration of serum PLP was found to be significantly associated with improved overall and colorectal cancer-specific survival after adjusting for various covariates. Higher PLP levels, particularly in the highest quartile group, were found to be associated with a 37% longer overall survival (HR_Q4 vs. Q1_, 0.63; 95% CI: 0.46, 0.87; *p* for trend = 0.008) and a 38% longer colorectal cancer-specific survival (HR_Q4 vs. Q1_, 0.62; 95% CI: 0.44, 0.87; *p* for trend = 0.006). In contrast, PAr was not significantly associated with overall survival (HR_Q4 vs. Q1_, 1.03; 95% CI: 0.75, 1.41; *p* for trend = 0.964) or colorectal cancer-specific survival (HR_Q4 vs. Q1_, 1.01; 95% CI: 0.72, 1.42; *p* for trend = 0.964) in the adjusted models (Table 2).

**Figure 2:**
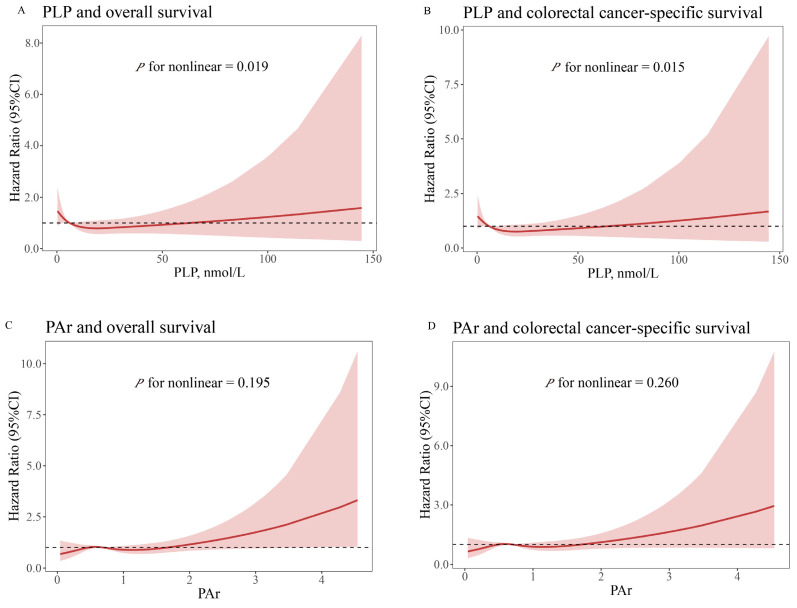
Multivariable-adjusted relationships between serum pyridoxal 5′-phosphate and PAr and survival outcome by the restricted cubic spline model. The solid red line represents the hazard ratio value, the shaded area represents the confidence interval, and the dashed line represents a hazard ratio value of 1. (**A**) PLP and overall survival. (**B**) PLP and colorectal cancer-specific survival. (**C**) PAr and overall survival. (**D**) PAr and colorectal cancer-specific survival. Cox proportional hazards regression model adjusted for age (continuous), sex (male, female), BMI (continuous), smoking (ever or never), drinking (regular or never), MET (continuous), total energy intake (continuous), protein intake (continuous), degree of differentiation (well-differentiated, moderately differentiated, poorly differentiated), surgery (yes or no), radiotherapy or chemotherapy (yes or no), and history of cancer in first-degree relatives (yes or no). Abbreviations: CI, confidence interval; PLP, pyridoxal 5′-phosphate; PAr, the ratio of PA over the combined concentrations of PLP and PL.

Further analyses, detailed in Figure 2, revealed a non-linear, reverse J-shaped association between serum PLP and both overall survival (*p* for non-linear = 0.019) and colorectal cancer-specific survival (*p* for non-linear = 0.015), as identified by the RCS analysis. Nevertheless, no statistically significant non-linear relationship was identified between PAr and either overall survival or colorectal cancer-specific survival (*p* for non-linear > 0.050).

### 3.3. Stratified and Sensitivity Analyses

Table 3 shows the association between serum vitamin B_6_ levels and survival outcomes in colorectal cancer patients stratified by factors such as sex, age, BMI, smoking status, alcohol drinking status, cancer stage, and tumor site. Interaction tests revealed that alcohol consumption significantly modified the relationship between serum PLP levels and colorectal cancer survival. Specifically, the association between serum PLP and both overall survival (*p* for interaction = 0.030) and colorectal cancer-specific survival (*p* for interaction = 0.031) varied by alcohol consumption. However, no significant interactions were found between PLP levels and survival outcomes across other factors such as sex, age, BMI, smoking status, cancer stage, or tumor site (all *p* for interaction > 0.05). Similarly, no significant interactions were found between PAr and survival outcomes across any of the examined variables (all *p* for interaction > 0.05). Specifically, the associations of serum PLP or PAr with overall survival and colorectal cancer-specific survival were consistent across subgroups such as sex, age, BMI, smoking status, cancer stage, and tumor site.

To address the potential impact of dietary intake and blood sample storage time on biomarker concentrations, we repeated our analyses, further adjusting for these factors in the fully adjusted model. Our results remained consistent. Sensitivity analyses confirmed that the associations of serum PLP levels with both overall and colorectal cancer-specific survival remained significant, even after adjusting for dietary vitamin B_6_ intake or the timing of blood-sample collection.

## 4. Discussion

This study demonstrated that colorectal cancer patients with elevated serum PLP levels experienced improved overall and colorectal cancer-specific survival. Additionally, the associations between serum PLP and survival were influenced by the drinking status of patients. No significant association was ascertained between serum PAr and either overall or colorectal cancer-specific survival. Sensitivity analyses confirmed the robustness of these findings.

To date, the existing research on this topic is relatively sparse, with only a limited number of studies having examined the relationship between circulating vitamin B_6_ concentrations and survival among colorectal cancer patients, with inconsistent findings reported [28,30,31]. Two of these studies, each involving fewer than 500 colorectal cancer cases, found no association between blood PLP levels and overall colorectal cancer survival [28,31], likely due to their small sample sizes. In contrast, a study involving 2031 patients with stage I-III colorectal cancer reported that elevated blood PLP levels were associated with improved overall survival [30], a finding consistent with our own results. Several factors influence PLP concentrations, including dietary intake, use of nutritional supplements, inflammatory status, serum levels of albumin and alkaline phosphatase, lifestyle habits, and exposure to food additives [44,45]. The variation in the estimated associations between PLP and cancer survival across different studies may be attributed to a number of factors, such as differences in study populations, methods of PLP measurement, sample sizes, follow-up periods, and vitamin B_6_ composition. Additionally, we found a non-linear relationship between serum PLP and survival in colorectal cancer patients, indicating there may be an optimal range for cancer patients. The findings imply that maintaining a specific concentration range of vitamins in the human body may be essential for ensuring optimal performance of the body’s normal physiological function [46,47]. In the process of DNA synthesis, 5,10-methylene tetrahydrofolate plays a role in the conversion of uracil to thymidylate. The regeneration of this enzyme is dependent on the presence of vitamin B_6_ [48]. There is a paucity of research examining the dose–response relationship between PLP and survival in colorectal cancer patients, and this study offers valuable insights in that area.

Our analysis did not reveal a significant association between low PAr and improved survival in colorectal cancer patients. To date, only one cohort study has investigated the role of PAr in colorectal cancer prognosis [30]. In contrast to our findings, the aforementioned study reported that lower PAr levels were associated with a 55% longer survival in adjusted models [30]. PA is a metabolite of vitamin B_6_ that is synthesized in the liver by PL and excreted primarily by the kidneys [45]. A study has demonstrated a correlation between elevated PA levels in elderly males and females and impaired kidney function [49]. This finding sheds light on the underlying mechanism contributing to elevated PAr levels in this population. As a result, stronger correlations between PAr and colorectal cancer survival, as reported by Holowatyj et al., were more likely to be observed. The discrepancy between our findings and the aforementioned study could be due to the timing of sample collection. Their study collected samples at least two weeks after treatment, whereas our samples were taken before diagnosis. Post-treatment vitamin B_6_ concentrations may have been influenced by medical interventions such as medications or surgery. In addition, differences in inflammatory status may explain the conflicting results, with the potential for collinearity between variables in the model that were adjusted for inflammation-related variables. This resulted in a reduction in the predictive value of vitamin B_6_ for the risk of mortality [50]. PAr, which is negatively correlated with PLP levels, serves as a reliable biomarker of systemic inflammation. It is useful for identifying individuals with elevated inflammatory status, as evidenced by markers like CRP and other inflammatory biomarkers [19,22,23,45,51,52]. Both PAr and inflammation may influence the progression of undiagnosed colorectal cancer, and the observed lack of associations in our study could be partially attributed to unmeasured inflammatory factors. The results of a nested case–control study indicated that PAr may be positively associated with tumor progression in colorectal cancer rather than with its initiation [23]. However, the role of PAr in colorectal cancer prognosis remains unclear, primarily due to limited data. Further study is required to gain a deeper understanding of the potential role of PAr in colorectal cancer prognosis.

Although the exact mechanism by which lower PLP levels contribute to poorer survival outcomes in patients with colorectal cancer is not fully understood, several possible mechanisms have been proposed. Deficiency of PLP may compromise DNA synthesis [53], alter methylation patterns [54], promote angiogenesis [55], increase oxidative stress [56], drive inflammation [57], and impair anti-tumor immunity [58]. It is also possible that the increased recruitment of the PLP cofactor for enzymes in the kynurenine pathway, the synthesis and catabolism of immunomodulatory sphingolipids, and serine hydroxymethylase supporting immune cell proliferation play a role in this process. This is supported by evidence from studies [59,60]. An additional cause of low plasma PLP is increased catabolism of vitamin B_6_ during inflammation [19]. Chronic inflammation can cause cellular DNA mutation and promote cancer development [61,62,63]. Pyridoxine supplementation significantly enhances the immune response in elderly individuals [64] or patients [65,66]. Therefore, it can be surmised that the association between vitamin B_6_ levels and mortality risk is mediated by its involvement in immune and inflammatory processes.

The present study revealed a significant interaction between serum PLP levels and alcohol consumption in relation to colorectal cancer survival. The findings suggest that alcohol consumption may elevate mortality in colorectal cancer patients, particularly those with low serum PLP levels. This interaction is consistent with the known biological mechanisms. First, alcohol consumption decreases vitamin B_6_ levels by interfering with methionine synthase [67], which plays a crucial role in vitamin B_6_ synthesis and absorption [68]. Second, chronic alcohol consumption decreases glutathione levels, which are synthesized from homocysteine through a process facilitated by two vitamin B_6_-dependent enzymes [67]. A reduction in glutathione levels might contribute to an increased vulnerability to DNA damage. It is also noteworthy that among patients over 50 years old or those who had never smoked, higher serum PLP levels were associated with longer overall and cancer-specific survival, although the interaction was not statistically significant. Additionally, our findings revealed that elevated serum PLP levels were linked to improved overall survival in males and in patients with overweight or obesity. Although the associations observed in some subgroups did not reach statistical significance due to the limited sample size, the results of the stratified analyses generally aligned with the main analysis.

A substantial amount of research has been conducted to determine the prognostic significance of various factors in colorectal cancer, aiming to identify effective prognostic markers such as epigenetic biomarkers, micro RNAs, and elements of the tumor microenvironment [69,70,71,72]. However, the transfer of these new biomarkers and targets from research to clinical practice requires larger-scale, multicenter trials to confirm their efficacy [70]. The importance of nutrition in cancer prognosis is gaining recognition in current cancer research [73], with nutrient supplementation emerging as a potential adjunctive treatment for colorectal cancer [74,75]. The underlying causes for the low circulating levels of PLP observed in colorectal cancer patients in our study are unclear; however, they may be associated with changes in intestinal microbiota and compromised metabolic function [76,77]. If colorectal cancer patients indeed have reduced absorption or metabolism of B vitamins, then oral supplementation or direct intravenous/intramuscular injection of B vitamins may effectively enhance their vitamin B_6_ levels [20,78]. In addition, non-pharmacological treatments for colorectal cancer often involve modifications in dietary habits and lifestyle changes [79,80]. Given the high prevalence of vitamin B_6_ deficiency among this patient group in China and the correlation between high PLP levels and improved survival rates, adjusting nutritional practices to address vitamin B_6_ deficiency could have significant implications for improving patient outcomes. Thus, the findings from this study provide valuable insights for developing effective nutritional strategies tailored for colorectal cancer patients [81].

The prospective design and the use of a highly accurate and precise LC-MS/MS-based method for measuring serum vitamin B_6_ levels are the two principal strengths of this study. Additionally, the study benefits from a considerable sample size, a lengthy follow-up period, detailed questionnaire data, and adjustments for various potential confounding factors. To our knowledge, this research is the first to demonstrate the association between serum vitamin B_6_ and survival outcomes in colorectal cancer patients in China. However, this study has some limitations. Firstly, the majority of participants in this study had serum PLP concentrations that were lower than those reported in previous studies. Nevertheless, it should be noted that the degree of the detection coefficients of variations in our data was relatively low (less than 15%), indicating that the serum PLP levels detected with the same method likely had minimal impact on the estimates. Secondly, vitamin B_6_ is present in foods like meat, which has been associated with an increased risk of cancer, while vegetables and fruits contain other nutrients that may have cancer-fighting properties. These food sources or compounds could potentially offset the effects of vitamin B_6_. Thus, it is a challenge to separate the independent impact of vitamin B_6_ on colorectal cancer prognosis from that of other foods or nutrients. Thirdly, as this is an observational study, residual confounding may have constrained our ability to establish causality. However, risk estimates remained unvaried following adjustment for diverse dietary and lifestyle factors, thereby suggesting that residual confounding is unlikely to account for our findings. Fourthly, serum vitamin B_6_ concentrations were only measured at baseline, and dynamic changes during the follow-up were not tracked. However, several cohort studies have shown that serum vitamin B_6_ levels tend to remain relatively stable over time [26]. Finally, the results of this study are based on the Chinese population, and caution is required when generalizing the findings.

## 5. Conclusions

This cohort study indicates that higher serum PLP levels may be associated with improved overall survival and colorectal cancer-specific survival. In contrast, PAr did not show a significant association with either overall survival or colorectal cancer-specific survival. Our findings underscore the potential importance of vitamin B_6_ in colorectal cancer prognosis and provide novel evidence suggesting that increased vitamin B_6_ intake could be beneficial for colorectal cancer patients.

## Figures and Tables

**Table 1:** Baseline characteristics of patients with colorectal cancer by the quartiles of serum pyridoxal 5’-phosphate in the Guangdong Colorectal Cancer Cohort.

Characteristics	Quartiles of Serum PLP (nmol/L) ^a^				*p*-Value ^b^
	Quartile 1 (*n* = 323)	Quartile 2 (*n* = 321)	Quartile 3 (*n* = 321)	Quartile 4 (*n* = 321)	
Sex (*n*, %)					1.000
Male	181 (56.04)	180 (56.07)	180 (56.07)	180 (56.07)	
Female	142 (43.96)	141 (43.93)	141 (43.93)	141 (43.93)	
Marital status (*n*, %)					0.094
Married	311 (96.28)	300 (93.46)	309 (96.26)	298 (92.83)	
Unmarried/divorced/widowed	12 (3.72)	21 (6.54)	12 (3.74)	23 (7.17)	
Residence (*n*, %)					0.279
Urban	211 (65.33)	192 (59.81)	211 (65.73)	213 (66.36)	
Rural	112 (34.67)	129 (40.19)	110 (34.27)	108 (33.64)	
Income, Yuan/month, (*n*, %)					0.995
less than 2000	49 (15.17)	47 (14.64)	46 (14.33)	43 (13.40)	
2001–5000	104 (32.20)	102 (31.78)	103 (32.09)	99 (30.84)	
5001–8000	90 (27.86)	99 (30.84)	97 (30.22)	97 (30.22)	
more than 8001	80 (24.77)	73 (22.74)	75 (23.36)	82 (25.55)	
Smoking status (*n*, %)					0.515
Never	187 (57.89)	196 (61.06)	189 (58.88)	203 (63.24)	
Ever	136 (42.11)	125 (38.94)	132 (41.12)	118 (36.76)	
Drinking, (*n*, %)					0.017
Never	280 (86.69)	271 (84.42)	250 (77.88)	272 (84.74)	
Regular	43 (13.31)	50 (15.58)	71 (22.12)	49 (15.26)	
BMI, kg/m^2^, (*n*, %)					0.211
<23.9	203 (62.85)	190 (59.19)	194 (60.44)	176 (54.83)	
≥23.9	120 (37.15)	131 (40.81)	127 (39.56)	145 (45.17)	
Physical activity, median (*P*_25_, *P*_75_), MET-h per week ^c^	28.88 (6.17, 52.50)	28.88 (10.31, 52.50)	26.25 (13.13, 52.50)	28.88 (11.25, 52.50)	0.940
Age at diagnosis in years, y, (*n*, %)					0.183
<50	78 (24.15)	85 (26.48)	69 (21.50)	92 (28.66)	
≥50	245 (75.85)	236 (73.52)	252 (78.50)	229 (71.34)	
Cancer stage (*n*, %) ^d^					<0.001
I	34 (10.53)	46 (14.33)	61 (19.00)	52 (16.20)	
II	112 (34.67)	113 (35.20)	111 (34.58)	104 (32.40)	
III	98 (30.34)	106 (33.02)	96 (29.91)	130 (40.50)	
IV	70 (21.67)	49 (15.26)	52 (16.20)	34 (10.59)	
Tumor site (*n*, %)					0.068
Colon	216 (66.87)	208 (64.80)	184 (57.32)	198 (61.68)	
Rectum	107 (33.13)	113 (35.20)	137 (42.68)	123 (38.32)	
Differentiation (*n*, %) ^e^					0.297
Well-differentiated	1 (0.31)	1 (0.31)	4 (1.25)	4 (1.25)	
Moderately differentiated	223 (69.04)	238 (74.14)	237 (73.83)	230 (71.65)	
Poorly differentiated	77 (23.84)	68 (21.18)	58 (18.07)	73 (22.74)	
Radiotherapy or chemotherapy (*n*, %)					0.656
No	114 (35.29)	108 (33.64)	120 (37.38)	106 (33.02)	
Yes	209 (64.71)	213 (66.36)	201 (62.62)	215 (66.98)	
Surgery (*n*, %)					0.239
No	22 (6.81)	14 (4.36)	12 (3.74)	13 (4.05)	
Yes	301 (93.19)	307 (95.64)	309 (96.26)	308 (95.95)	
History of cancer in first-degree relatives (*n*, %)					0.753
No	279 (86.38)	274 (85.36)	283 (88.16)	280 (87.23)	
Yes	44 (13.62)	47 (14.64)	38 (11.84)	41 (12.77)	
PL (nmol/L), median (*P*_25_, *P*_75_)	10.78 (6.70, 15.62)	10.92 (7.62, 15.19)	10.97 (7.78, 16.84)	16.00 (9.31, 28.70)	<0.001
PA (nmol/L), median (*P*_25_, *P*_75_)	11.22 (7.62, 15.73)	12.68 (9.63, 18.19)	12.99 (9.24, 18.71)	15.61 (11.15, 28.60)	<0.001
PAr, median (*P*_25_, *P*_75_)	0.84 (0.58, 1.23)	0.83 (0.58, 1.14)	0.67 (0.47, 0.94)	0.49 (0.33, 0.67)	<0.001
Dietary intake, median (*P*_25_, *P*_75_) ^f^					
Total energy (kcal/day)	1544.15 (1263.22, 1817.55)	1524.82 (1278.14, 1864.33)	1519.02 (1250.05, 1858.67)	1424.64 (1152.41, 1752.29)	0.011
Protein (g/day)	65.67 (55.35, 79.31)	66.90 (53.58, 78.52)	63.30 (52.42, 77.32)	60.05 (49.28, 75.61)	0.004
Vitamin B_2_ (mg/day)	0.83 (0.71, 0.98)	0.83 (0.69, 0.98)	0.82 (0.68, 0.97)	0.82 (0.70, 1.00)	0.456
Vitamin B_6_ (mg/day)	0.83 (0.71, 0.97)	0.83 (0.71, 0.93)	0.80 (0.67, 0.94)	0.80 (0.69, 0.92)	0.062
Folate (μg/day)	208.59 (180.26, 240.70)	200.84 (178.58, 237.52)	205.38 (172.86, 242.00)	208.70 (176.69, 247.09)	0.397
Vitamin B_12_ (mg/day)	1.67 (1.20, 2.15)	1.65 (1.16, 2.36)	1.62 (1.16, 2.25)	1.65 (1.10, 2.37)	0.939

Abbreviations: PLP, pyridoxal 5′-phosphate; BMI, body mass index; MET, metabolic equivalent task; PL, pyridoxal; PA, 4-pyridoxic acid; PAr, the ratio of PA over the combined concentrations of PLP and PL. ^a^ Quartile ranges of serum PLP and PL were <3.11, 3.11 to 5.86, 5.86 to 10.78, and ≥10.78 nmol/L in men and <3.33, 3.33 to 6.27, 6.27 to 11.59, and ≥11.59 nmol/L in women. ^b^ Chi-square tests or Kruskal-Wallis tests, *p*-value < 0.05 were considered statistically significant. ^c^ Six patients had unknown information on MET. ^d^ Eighteen patients had unknown information on cancer stage. ^e^ Seventy-two patients had other or unknown degree of differentiation. ^f^ Consumption was adjusted for total energy intake using the regression residual method.

**Table 2:** Hazard ratio (95% confidence interval) for overall survival and overall survival and colorectal cancer-specific survival according to quartiles of serum pyridoxal 5’-phosphate and PAr in the Guangdong Colorectal Cancer Cohort.

	Death/Number	Crude Model	Model 1 ^b^	Model 2 ^c^
Overall survival				
PLP, nmol/L				
Quartile 1	96/323	1.00	1.00	1.00
Quartile 2	82/321	0.80 (0.60–1.08)	0.79 (0.59–1.06)	0.81 (0.60–1.09)
Quartile 3	84/321	0.79 (0.59–1.06)	0.78 (0.58–1.05)	0.81 (0.60–1.09)
Quartile 4	69/321	0.65 (0.47–0.88)	0.66 (0.48–0.91)	0.63 (0.46–0.87)
*p* for trend ^a^		0.004	0.021	0.008
PAr				
Quartile 1	73/313	1.00	1.00	1.00
Quartile 2	85/321	1.14 (0.83–1.56)	1.10 (0.81–1.51)	1.07 (0.78–1.46)
Quartile 3	86/323	1.17 (0.85–1.59)	1.13 (0.83–1.55)	1.10 (0.80–1.51)
Quartile 4	87/329	1.15 (0.84–1.57)	1.07 (0.78–1.47)	1.03 (0.75–1.41)
*p* for trend ^a^		0.293	0.797	0.964
Colorectal cancer-specific survival				
PLP, nmol/L				
Quartile 1	85/323	1.00	1.00	1.00
Quartile 2	76/321	0.84 (0.62–1.15)	0.83 (0.61–1.13)	0.85 (0.62–1.16)
Quartile 3	72/321	0.77 (0.56–1.06)	0.77 (0.56–1.05)	0.79 (0.58–1.09)
Quartile 4	60/321	0.64 (0.46–0.89)	0.65 (0.47–0.91)	0.62 (0.44–0.87)
*p* for trend ^a^		0.004	0.016	0.006
PAr				
Quartile 1	65/313	1.00	1.00	1.00
Quartile 2	75/321	1.14 (0.82–1.58)	1.11 (0.80–1.55)	1.07 (0.76–1.49)
Quartile 3	78/323	1.19 (0.86–1.66)	1.17 (0.84–1.63)	1.12 (0.80–1.56)
Quartile 4	75/329	1.13 (0.81–1.58)	1.06 (0.75–1.48)	1.01 (0.72–1.42)
*p* for trend ^a^		0.398	0.892	0.964

^a^ Test for linear trend was based on the median values for each quartile of serum concentration. ^b^ Cox proportional hazards regression model adjusted for age (continuous), sex (male, female), BMI (continuous), smoking (ever or never), drinking (regular or never), MET (continuous), total energy intake (continuous), and protein intake (continuous). ^c^ Cox proportional hazards regression model additionally adjusted for differentiation (well-differentiated, moderately differentiated, poorly differentiated), surgery (yes or no), radiotherapy or chemotherapy (yes or no), and history of cancer in first-degree relatives (yes or no). Abbreviations: PLP, pyridoxal 5′-phosphate; PAr, the ratio of PA over the combined concentrations of PLP and PL.

**Table 3:** Hazard ratios (95% CI) for survival outcomes by pyridoxal 5’-phosphate and PAr stratified by selected covariates in the Guangdong Colorectal Cancer Cohort Study.

	Overall Survival				Colorectal Cancer-Specific Survival			
	Quartile 1	Quartile 4 ^a^	*p* for Trend ^b^	*p* for Interaction ^c^	Quartile 1	Quartile 4 ^a^	*p* for Trend ^b^	*p* for Interaction ^c^
PLP, nmol/L								
Sex				0.468				0.581
Male	1.00	0.64 (0.43–0.94)	0.062		1.00	0.66 (0.44–1.01)	0.088	
Female	1.00	0.66 (0.38–1.15)	0.077		1.00	0.61 (0.34–1.08)	0.044	
Age				0.674				0.705
<50	1.00	0.68 (0.37–1.24)	0.436		1.00	0.56 (0.29–1.07)	0.196	
≥50	1.00	0.62 (0.43–0.89)	0.011		1.00	0.65 (0.44–0.97)	0.023	
BMI				0.611				0.888
<23.9	1.00	0.72 (0.48–1.08)	0.106		1.00	0.65 (0.42–1.01)	0.044	
≥23.9	1.00	0.50 (0.30–0.83)	0.026		1.00	0.58 (0.34–0.99)	0.080	
Smoking status				0.098				0.118
Ever	1.00	0.84 (0.54–1.31)	0.748		1.00	0.9 (0.55–1.460)	0.855	
Never	1.00	0.51 (0.32–0.80)	0.002		1.00	0.48 (0.29–0.77)	0.001	
Drinking status				0.030				0.031
Regular	1.00	0.57 (0.24–1.32)	0.181		1.00	0.72 (0.27–1.90)	0.261	
Never	1.00	0.67 (0.48–0.94)	0.029		1.00	0.64 (0.45–0.92)	0.020	
Cancer stage				0.717				0.581
I–III	1.00	0.80 (0.51–1.26)	0.323		1.00	0.74 (0.45–1.22)	0.169	
IV	1.00	1.08 (0.67–1.74)	0.637		1.00	0.95 (0.60–1.50)	0.433	
Tumor site				0.340				0.501
Colon	1.00	0.71 (0.47–1.07)	0.174		1.00	0.68 (0.44–1.06)	0.126	
Rectum	1.00	0.70 (0.41–1.16)	0.132		1.00	0.74 (0.42–1.28)	0.177	
PAr								
Sex				0.811				0.769
Male	1.00	0.95 (0.65–1.40)	0.790		1.00	0.92 (0.61–1.39)	0.627	
Female	1.00	1.11 (0.62–2.00)	0.936		1.00	1.14 (0.62–2.11)	0.782	
Age				0.889				0.797
<50	1.00	1.08 (0.53–2.20)	0.742		1.00	1.37 (0.65–2.90)	0.427	
≥50	1.00	1.06 (0.74–1.52)	0.890		1.00	0.97 (0.66–1.42)	0.812	
BMI				0.838				0.943
<23.9	1.00	1.08 (0.72–1.62)	0.913		1.00	1.12 (0.74–1.71)	0.705	
≥23.9	1.00	1.00 (0.59–1.69)	0.769		1.00	0.88 (0.49–1.58)	0.751	
Smoking status				0.452				0.425
Ever	1.00	0.94 (0.61–1.44)	0.995		1.00	0.85 (0.54–1.36)	0.681	
Never	1.00	1.03 (0.64–1.68)	0.645		1.00	1.11 (0.66–1.85)	0.840	
Drinking status				0.882				0.945
Regular	1.00	0.93 (0.49–1.80)	0.989		1.00	0.84 (0.42–1.71)	0.695	
Never	1.00	1.04 (0.72–1.50)	0.971		1.00	e	e	
Cancer stage				0.717				0.593
I–III	1.00	0.89 (0.57–1.41)	0.492		1.00	0.88 (0.52–1.47)	0.418	
IV	1.00	0.89 (0.55–1.42)	0.911		1.00	e	0.825	
Tumor site				0.819				0.770
Colon	1.00	1.04 (0.68–1.59)	0.800		1.00	0.93 (0.59–1.47)	0.455	
Rectum	1.00	0.88 (0.53–1.47)	0.699		1.00	0.98 (0.58–1.68)	0.972	

^a^ Cox proportional hazards regression model adjusted for age (continuous), sex (male, female), BMI (continuous), smoking (ever or never), drinking (regular or never), MET (continuous), total energy intake (continuous), protein intake (continuous), differentiation (well-differentiated, moderately differentiated, poorly differentiated), surgery (yes or no), radiotherapy or chemotherapy (yes or no), and history of cancer in first-degree relatives (yes or no). ^b^ The linear trend was conducted using the median values for each quartile of serum concentration. ^c^ The interaction term’s significance was assessed using the Wald method to calculate the *p*-value for interaction, which tested the multiplicative interaction between serum vitamin B_6_ levels and the respective stratified variable. Abbreviations: PLP, pyridoxal 5′-phosphate; PAr, the ratio of PA over the combined concentrations of PLP and PL.

## Data Availability

The data that support the findings of our study are available from the corresponding author upon reasonable request. The data are not publicly available due to technical limitations.

## References

[1] Sung H., Ferlay J., Siegel R.L., Laversanne M., Soerjomataram I., Jemal A., Bray F. (2021). Global Cancer Statistics 2020: GLOBOCAN Estimates of Incidence and Mortality Worldwide for 36 Cancers in 185 Countries. CA Cancer J. Clin..

[2] GBD 2019 Colorectal Cancer Collaborators (2022). Global, regional, and national burden of colorectal cancer and its risk factors, 1990–2019: A systematic analysis for the Global Burden of Disease Study 2019. Lancet Gastroenterol. Hepatol..

[3] Murphy C.C., Zaki T.A. (2024). Changing epidemiology of colorectal cancer—Birth cohort effects and emerging risk factors. Nat. Rev. Gastroenterol. Hepatol..

[4] Baidoun F., Elshiwy K., Elkeraie Y., Merjaneh Z., Khoudari G., Sarmini M.T., Gad M., Al-Husseini M., Saad A. (2021). Colorectal Cancer Epidemiology: Recent Trends and Impact on Outcomes. Curr. Drug Targets.

[5] Biller L.H., Schrag D. (2021). Diagnosis and Treatment of Metastatic Colorectal Cancer: A Review. JAMA.

[6] Franco C.N., Seabrook L.J., Nguyen S.T., Leonard J.T., Albrecht L.V. (2022). Simplifying the B Complex: How Vitamins B6 and B9 Modulate One Carbon Metabolism in Cancer and Beyond. Metabolites.

[7] Sedlak J.C., Yilmaz Ö.H., Roper J. (2023). Metabolism and Colorectal Cancer. Annu. Rev. Pathol..

[8] Jeong Y.J., Rogers T.J., Anderson C.E., Lien E.C. (2023). Tumor lipid metabolism: A mechanistic link between diet and cancer progression. Curr. Opin. Biotechnol..

[9] Shah U.A., Iyengar N.M. (2022). Plant-Based and Ketogenic Diets as Diverging Paths to Address Cancer: A Review. JAMA Oncol..

[10] Zitvogel L., Pietrocola F., Kroemer G. (2017). Nutrition, inflammation and cancer. Nat. Immunol..

[11] Nasir A., Bullo M.M.H., Ahmed Z., Imtiaz A., Yaqoob E., Jadoon M., Ahmed H., Afreen A., Yaqoob S. (2020). Nutrigenomics: Epigenetics and cancer prevention: A comprehensive review. Crit. Rev. Food Sci. Nutr..

[12] Stidley C.A., Picchi M.A., Leng S., Willink R., Crowell R.E., Flores K.G., Kang H., Byers T., Gilliland F.D., Belinsky S.A. (2010). Multivitamins, folate, and green vegetables protect against gene promoter methylation in the aerodigestive tract of smokers. Cancer Res..

[13] Hardy T.M., Tollefsbol T.O. (2011). Epigenetic diet: Impact on the epigenome and cancer. Epigenomics.

[14] Duthie S.J. (2011). Folate and cancer: How DNA damage, repair and methylation impact on colon carcinogenesis. J. Inherit. Metab. Dis..

[15] Kawakita D., Matsuo K., Sato F., Oze I., Hosono S., Ito H., Watanabe M., Yatabe Y., Hanai N., Hasegawa Y. (2012). Association between dietary folate intake and clinical outcome in head and neck squamous cell carcinoma. Ann. Oncol..

[16] Mikkelsen K., Stojanovska L., Apostolopoulos V. (2016). The Effects of Vitamin B in Depression. Curr. Med. Chem..

[17] Bassett J.K., Brinkman M.T., Dugué P.A., Ueland P.M., Midttun Ø., Ulvik A., Bolton D., Southey M.C., English D.R., Milne R.L. (2019). Circulating concentrations of B group vitamins and urothelial cell carcinoma. Int. J. Cancer.

[18] Leklem J.E. (1990). Vitamin B-6: A status report. J. Nutr..

[19] Ulvik A., Midttun Ø., Pedersen E.R., Eussen S.J., Nygård O., Ueland P.M. (2014). Evidence for increased catabolism of vitamin B-6 during systemic inflammation. Am. J. Clin. Nutr..

[20] Ryan K.M., Allers K.A., Harkin A., McLoughlin D.M. (2020). Blood plasma B vitamins in depression and the therapeutic response to electroconvulsive therapy. Brain Behav. Immun. Health.

[21] Zuo H., Ueland P.M., Midttun Ø., Vollset S.E., Tell G.S., Theofylaktopoulou D., Travis R.C., Boutron-Ruault M.C., Fournier A., Severi G. (2018). Results from the European Prospective Investigation into Cancer and Nutrition Link Vitamin B6 Catabolism and Lung Cancer Risk. Cancer Res..

[22] Zuo H., Tell G.S., Ueland P.M., Nygård O., Vollset S.E., Midttun Ø., Meyer K., Ulvik A. (2018). The PAr index, an indicator reflecting altered vitamin B-6 homeostasis, is associated with long-term risk of stroke in the general population: The Hordaland Health Study (HUSK). Am. J. Clin. Nutr..

[23] Gylling B., Myte R., Schneede J., Hallmans G., Häggström J., Johansson I., Ulvik A., Ueland P.M., Van Guelpen B., Palmqvist R. (2017). Vitamin B-6 and colorectal cancer risk: A prospective population-based study using 3 distinct plasma markers of vitamin B-6 status. Am. J. Clin. Nutr..

[24] Neuhouser M.L., Cheng T.Y., Beresford S.A., Brown E., Song X., Miller J.W., Zheng Y., Thomson C.A., Shikany J.M., Vitolins M.Z. (2015). Red blood cell folate and plasma folate are not associated with risk of incident colorectal cancer in the Women’s Health Initiative observational study. Int. J. Cancer.

[25] Weinstein S.J., Albanes D., Selhub J., Graubard B., Lim U., Taylor P.R., Virtamo J., Stolzenberg-Solomon R. (2008). One-carbon metabolism biomarkers and risk of colon and rectal cancers. Cancer Epidemiol. Biomark. Prev..

[26] Eussen S.J., Vollset S.E., Hustad S., Midttun Ø., Meyer K., Fredriksen A., Ueland P.M., Jenab M., Slimani N., Boffetta P. (2010). Plasma vitamins B2, B6, and B12, and related genetic variants as predictors of colorectal cancer risk. Cancer Epidemiol. Biomark. Prev..

[27] Dray X., Boutron-Ruault M.C., Bertrais S., Sapinho D., Benhamiche-Bouvier A.M., Faivre J. (2003). Influence of dietary factors on colorectal cancer survival. Gut.

[28] Leung E.Y., Roxburgh C.S., Talwar D., O’Reilly D.S., McKee R.F., Horgan P.G., McMillan D.C. (2012). The relationships between plasma and red cell vitamin B2 and B6 concentrations and the systemic and local inflammatory responses in patients with colorectal cancer. Nutr. Cancer.

[29] Lochhead P., Nishihara R., Qian Z.R., Mima K., Cao Y., Sukawa Y., Kim S.A., Inamura K., Zhang X., Wu K. (2015). Postdiagnostic intake of one-carbon nutrients and alcohol in relation to colorectal cancer survival. Am. J. Clin. Nutr..

[30] Holowatyj A.N., Ose J., Gigic B., Lin T., Ulvik A., Geijsen A., Brezina S., Kiblawi R., van Roekel E.H., Baierl A. (2022). Higher vitamin B6 status is associated with improved survival among patients with stage I-III colorectal cancer. Am. J. Clin. Nutr..

[31] Je Y., Lee J.E., Ma J., Zhang X., Cho E., Rosner B., Selhub J., Fuchs C.S., Meyerhardt J., Giovannucci E. (2013). Prediagnostic plasma vitamin B6 (pyridoxal 5′-phosphate) and survival in patients with colorectal cancer. Cancer Causes Control.

[32] Zhong X., Fang Y.J., Pan Z.Z., Li B., Wang L., Zheng M.C., Chen Y.M., Zhang C.X. (2013). Dietary fat, fatty acid intakes and colorectal cancer risk in Chinese adults: A case-control study. Eur. J. Cancer Prev..

[33] Ma T., Tu K., Ou Q., Fang Y., Zhang C. (2023). Comparing the Associations of Dietary Patterns Identified through Principal Component Analysis and Cluster Analysis with Colorectal Cancer Risk: A Large Case-Control Study in China. Nutrients.

[34] Meisser Redeuil K., Longet K., Bénet S., Munari C., Campos-Giménez E. (2015). Simultaneous quantification of 21 water soluble vitamin circulating forms in human plasma by liquid chromatography-mass spectrometry. J. Chromatogr. A.

[35] Midttun O., Hustad S., Solheim E., Schneede J., Ueland P.M. (2005). Multianalyte quantification of vitamin B6 and B2 species in the nanomolar range in human plasma by liquid chromatography-tandem mass spectrometry. Clin. Chem..

[36] Xu L., Fang Y.J., Che M.M., Abulimiti A., Huang C.Y., Zhang C.X. (2022). Association of Serum Pyridoxal-5′-Phosphate, Pyridoxal, and PAr with Colorectal Cancer Risk: A Large-Scale Case-Control Study. Nutrients.

[37] Xu L., Wu Q.X., Li X., Fang Y.J., Zhou R.L., Che M.M., Ma T., Zhang C.X. (2022). Serum flavin mononucleotide but not riboflavin is inversely associated with the risk of colorectal cancer. Food Funct..

[38] Zhang C.X., Ho S.C. (2009). Validity and reproducibility of a food frequency Questionnaire among Chinese women in Guangdong province. Asia Pac. J. Clin. Nutr..

[39] Yang Y.X., Wang G.Y., Pan X.C. (2002). China Food Composition Table.

[40] Willett W., Stampfer M.J. (1986). Total energy intake: Implications for epidemiologic analyses. Am. J. Epidemiol..

[41] Ainsworth B.E., Haskell W.L., Whitt M.C., Irwin M.L., Swartz A.M., Strath S.J., O’Brien W.L., Bassett D.R., Schmitz K.H., Emplaincourt P.O. (2000). Compendium of physical activities: An update of activity codes and MET intensities. Med. Sci. Sports Exerc..

[42] Ainsworth B.E., Haskell W.L., Herrmann S.D., Meckes N., Bassett D.R., Tudor-Locke C., Greer J.L., Vezina J., Whitt-Glover M.C., Leon A.S. (2011). 2011 Compendium of Physical Activities: A second update of codes and MET values. Med. Sci. Sports Exerc..

[43] Rumgay H., Murphy N., Ferrari P., Soerjomataram I. (2021). Alcohol and Cancer: Epidemiology and Biological Mechanisms. Nutrients.

[44] Midttun Ø., Theofylaktopoulou D., McCann A., Fanidi A., Muller D.C., Meyer K., Ulvik A., Zheng W., Shu X.O., Xiang Y.B. (2017). Circulating concentrations of biomarkers and metabolites related to vitamin status, one-carbon and the kynurenine pathways in US, Nordic, Asian, and Australian populations. Am. J. Clin. Nutr..

[45] Ueland P.M., Ulvik A., Rios-Avila L., Midttun Ø., Gregory J.F. (2015). Direct and Functional Biomarkers of Vitamin B6 Status. Annu. Rev. Nutr..

[46] Bjelakovic G., Gluud C. (2011). Vitamin and mineral supplement use in relation to all-cause mortality in the Iowa Women’s Health Study. Arch. Intern. Med..

[47] Vrolijk M.F., Opperhuizen A., Jansen E., Hageman G.J., Bast A., Haenen G. (2017). The vitamin B6 paradox: Supplementation with high concentrations of pyridoxine leads to decreased vitamin B6 function. Toxicol. Vitr..

[48] Selhub J. (2002). Folate, vitamin B12 and vitamin B6 and one carbon metabolism. J. Nutr. Health Aging.

[49] Bates C.J., Pentieva K.D., Prentice A. (1999). An appraisal of vitamin B6 status indices and associated confounders, in young people aged 4-18 years and in people aged 65 years and over, in two national British surveys. Public Health Nutr..

[50] Wang P., Huang J., Xue F., Abuduaini M., Tao Y., Liu H. (2024). Associations of serum vitamin B6 status with the risks of cardiovascular, cancer, and all-cause mortality in the elderly. Front. Immunol..

[51] Kwak H.K., Hansen C.M., Leklem J.E., Hardin K., Shultz T.D. (2002). Improved vitamin B-6 status is positively related to lymphocyte proliferation in young women consuming a controlled diet. J. Nutr..

[52] Chiang E.P., Smith D.E., Selhub J., Dallal G., Wang Y.C., Roubenoff R. (2005). Inflammation causes tissue-specific depletion of vitamin B6. Arthritis Res. Ther..

[53] Ames B.N. (2001). DNA damage from micronutrient deficiencies is likely to be a major cause of cancer. Mutat. Res..

[54] Anderson O.S., Sant K.E., Dolinoy D.C. (2012). Nutrition and epigenetics: An interplay of dietary methyl donors, one-carbon metabolism and DNA methylation. J. Nutr. Biochem..

[55] Matsubara K., Komatsu S., Oka T., Kato N. (2003). Vitamin B6-mediated suppression of colon tumorigenesis, cell proliferation, and angiogenesis (review). J. Nutr. Biochem..

[56] Komatsu S., Watanabe H., Oka T., Tsuge H., Kat N. (2002). Dietary vitamin B6 suppresses colon tumorigenesis, 8-hydroxyguanosine, 4-hydroxynonenal, and inducible nitric oxide synthase protein in azoxymethane-treated mice. J. Nutr. Sci. Vitaminol..

[57] Shen J., Lai C.Q., Mattei J., Ordovas J.M., Tucker K.L. (2010). Association of vitamin B-6 status with inflammation, oxidative stress, and chronic inflammatory conditions: The Boston Puerto Rican Health Study. Am. J. Clin. Nutr..

[58] Bargiela D., Cunha P.P., Veliça P., Foskolou I.P., Barbieri L., Rundqvist H., Johnson R.S. (2022). Vitamin B6 Metabolism Determines T Cell Anti-Tumor Responses. Front. Immunol..

[59] Sakakeeny L., Roubenoff R., Obin M., Fontes J.D., Benjamin E.J., Bujanover Y., Jacques P.F., Selhub J. (2012). Plasma pyridoxal-5-phosphate is inversely associated with systemic markers of inflammation in a population of U.S. adults. J. Nutr..

[60] Paul L., Ueland P.M., Selhub J. (2013). Mechanistic perspective on the relationship between pyridoxal 5′-phosphate and inflammation. Nutr. Rev..

[61] Elinav E., Nowarski R., Thaiss C.A., Hu B., Jin C., Flavell R.A. (2013). Inflammation-induced cancer: Crosstalk between tumours, immune cells and microorganisms. Nat. Rev. Cancer.

[62] Colotta F., Allavena P., Sica A., Garlanda C., Mantovani A. (2009). Cancer-related inflammation, the seventh hallmark of cancer: Links to genetic instability. Carcinogenesis.

[63] Federico A., Morgillo F., Tuccillo C., Ciardiello F., Loguercio C. (2007). Chronic inflammation and oxidative stress in human carcinogenesis. Int. J. Cancer.

[64] Talbott M.C., Miller L.T., Kerkvliet N.I. (1987). Pyridoxine supplementation: Effect on lymphocyte responses in elderly persons. Am. J. Clin. Nutr..

[65] Casciato D.A., McAdam L.P., Kopple J.D., Bluestone R., Goldberg L.S., Clements P.J., Knutson D.W. (1984). Immunologic abnormalities in hemodialysis patients: Improvement after pyridoxine therapy. Nephron.

[66] Cheng C.H., Chang S.J., Lee B.J., Lin K.L., Huang Y.C. (2006). Vitamin B6 supplementation increases immune responses in critically ill patients. Eur. J. Clin. Nutr..

[67] Larsson S.C., Giovannucci E., Wolk A. (2005). Vitamin B6 intake, alcohol consumption, and colorectal cancer: A longitudinal population-based cohort of women. Gastroenterology.

[68] Yasuda H., Hatano T., Honda T., Tsutsui M., Hattori N., Ando M., Komatsu N. (2022). Vitamin B6 Deficiency Anemia Attributed to Levodopa/Carbidopa Intestinal Gel Therapy for Parkinson’s Disease: A Diagnostic Pitfall for Myelodysplastic Syndrome with Ring Sideroblasts. Intern. Med..

[69] Gallo G., Vescio G., De Paola G., Sammarco G. (2021). Therapeutic Targets and Tumor Microenvironment in Colorectal Cancer. J. Clin. Med..

[70] Luo X.J., Zhao Q., Liu J., Zheng J.B., Qiu M.Z., Ju H.Q., Xu R.H. (2021). Novel Genetic and Epigenetic Biomarkers of Prognostic and Predictive Significance in Stage II/III Colorectal Cancer. Mol. Ther..

[71] Cao Q., Tian Y., Deng Z., Yang F., Chen E. (2024). Epigenetic Alteration in Colorectal Cancer: Potential Diagnostic and Prognostic Implications. Int. J. Mol. Sci..

[72] Giannopoulou N., Constantinou C. (2023). Recent Developments in Diagnostic and Prognostic Biomarkers for Colorectal Cancer: A Narrative Review. Oncology.

[73] Chan D.S.M., Cariolou M., Markozannes G., Balducci K., Vieira R., Kiss S., Becerra-Tomás N., Aune D., Greenwood D.C., González-Gil E.M. (2024). Post-diagnosis dietary factors, supplement use and colorectal cancer prognosis: A Global Cancer Update Programme (CUP Global) systematic literature review and meta-analysis. Int. J. Cancer.

[74] Henriksen H.B., Ræder H., Bøhn S.K., Paur I., Kværner A.S., Billington S., Eriksen M.T., Wiedsvang G., Erlund I., Færden A. (2017). The Norwegian dietary guidelines and colorectal cancer survival (CRC-NORDIET) study: A food-based multicentre randomized controlled trial. BMC Cancer.

[75] Ravasco P., Monteiro-Grillo I., Camilo M. (2012). Individualized nutrition intervention is of major benefit to colorectal cancer patients: Long-term follow-up of a randomized controlled trial of nutritional therapy. Am. J. Clin. Nutr..

[76] Rosenberg J., Ischebeck T., Commichau F.M. (2017). Vitamin B6 metabolism in microbes and approaches for fermentative production. Biotechnol. Adv..

[77] Feng Z., Hua J., Guo F., Liu Z., Zhao Y., Wu W. (2023). A retrospective analysis of vitamin B6 deficiency and associated changes of gut microbes in Crohn’s disease. Eur. J. Clin. Nutr..

[78] Zheng C., Ge Q., Luo C., Hu L., Shen Y., Xue Q. (2022). Enteral nutrition improves the prognosis and immune nutritional status of patients in the cardiothoracic surgery recovery unit: A propensity score-matched analysis. Clin. Nutr..

[79] Martínez-Montoro J.I., Martínez-Sánchez M.A., Balaguer-Román A., Gil-Martínez J., Mesa-López M.J., Egea-Valenzuela J., Ruiz-Alcaraz A.J., Queipo-Ortuño M.I., Ferrer M., Fernández-García J.C. (2022). Dietary modulation of gut microbiota in patients with colorectal cancer undergoing surgery: A review. Int. J. Surg..

[80] Duncan M., Moschopoulou E., Herrington E., Deane J., Roylance R., Jones L., Bourke L., Morgan A., Chalder T., Thaha M.A. (2017). Review of systematic reviews of non-pharmacological interventions to improve quality of life in cancer survivors. BMJ Open.

[81] Chen Y., Liu B.L., Shang B., Chen A.S., Liu S.Q., Sun W., Yin H.Z., Yin J.Q., Su Q. (2011). Nutrition support in surgical patients with colorectal cancer. World J. Gastroenterol..

